# Demographic Predictors of Peanut, Tree Nut, Fish, Shellfish, and Sesame Allergy in Canada

**DOI:** 10.1155/2012/858306

**Published:** 2011-12-01

**Authors:** M. Ben-Shoshan, D. W. Harrington, L. Soller, J. Fragapane, L. Joseph, Y. St. Pierre, S. B. Godefroy, S. J. Elliott, A. E. Clarke

**Affiliations:** ^1^Division of Pediatric Allergy and Clinical Immunology, Department of Pediatrics, Montreal Children's Hospital, 2300 Tupper Street, Montreal, QC, Canada H3H 1P3; ^2^School of Geography and Earth Sciences, McMaster University, Hamilton, ON, Canada L8S 4L8; ^3^Division of Clinical Epidemiology, Department of Medicine, McGill University Health Center, Canada; ^4^Departments of Epidemiology and Biostatistics, McGill University, QC, Canada H3A 3R1; ^5^Food Directorate, Health Canada, Ottawa, ON, Canada K1A 0K9; ^6^Applied Health Sciences, University of Waterloo, ON, Canada N2L 3G1; ^7^Division of Allergy and Clinical Immunology, Department of Medicine, McGill University Health Center, QC, Canada H3H 2R9

## Abstract

*Background*. Studies suggest that the rising prevalence of food allergy during recent decades may have stabilized. Although genetics undoubtedly contribute to the emergence of food allergy, it is likely that other factors play a crucial role in mediating such short-term changes. *Objective*. To identify potential demographic predictors of food allergies. *Methods*. We performed a cross-Canada, random telephone survey. Criteria for food allergy were self-report of convincing symptoms and/or physician diagnosis of allergy. Multivariate logistic regressions were used to assess potential determinants. *Results*. Of 10,596 households surveyed in 2008/2009, 3666 responded, representing 9667 individuals. Peanut, tree nut, and sesame allergy were more common in children (odds ratio (OR) 2.24 (95% CI, 1.40, 3.59), 1.73 (95% CI, 1.11, 2.68), and 5.63 (95% CI, 1.39, 22.87), resp.) while fish and shellfish allergy were less common in children (OR 0.17 (95% CI, 0.04, 0.72) and 0.29 (95% CI, 0.14, 0.61)). Tree nut and shellfish allergy were less common in males (OR 0.55 (95% CI, 0.36, 0.83) and 0.63 (95% CI, 0.43, 0.91)). Shellfish allergy was more common in urban settings (OR 1.55 (95% CI, 1.04, 2.31)). There was a trend for most food allergies to be more prevalent in the more educated (tree nut OR 1.90 (95% CI, 1.18, 3.04)) and less prevalent in immigrants (shellfish OR 0.49 (95% CI, 0.26, 0.95)), but wide CIs preclude definitive conclusions for most foods. *Conclusions*. Our results reveal that in addition to age and sex, place of residence, socioeconomic status, and birth place may influence the development of food allergy.

## 1. Introduction

Among adults worldwide, 7.7% (Iceland) to 24.6% (US) are sensitized to food allergens [1]. Foods are the most common triggers for anaphylaxis, accounting for 33.2% to 56% of all cases [2, 3] with peanut, tree nut, fish, and shellfish responsible for the majority of fatal reactions [4]. Studies suggest an increasing prevalence of food allergies in the past two decades [5, 6], with a recent stabilization in developed countries [7, 8]. Although genetic factors undoubtedly contribute to the development of food allergies [9], it is evident that they are not fully responsible for these relatively short-term temporal trends in prevalence. Further, recent reports suggest that populations with similar genetic backgrounds may have different rates of food allergy, possibly due to different dietary habits [10], and alternatively, populations with different genetic backgrounds may have the same relative prevalence of food allergies [1]. It is evident that the development of food allergy results from an interplay of genetic, environmental, and demographic factors. However, little is known about which demographic factors are associated with food allergy. In the SCAAALAR study (Surveying Canadians to Assess the Prevalence of Common Food Allergies and Attitudes towards Food LAbelling and Risk) launched in 2008, we determined the nationwide prevalence of peanut, tree nut, fish, shellfish, and sesame allergy [11]. In this manuscript, we evaluate potential demographic factors that may influence the prevalence of these potentially severe food allergies in the SCAAALAR population.

## 2. Methods

As described in detail elsewhere [11], households in the ten Canadian provinces were chosen by purchasing, from Info-Direct, a random selection of telephone numbers and their accompanying addresses from the electronic white pages. Interviews were conducted from May 2008 to March 2009 by trained interviewers from either McGill (Montreal, Quebec) or McMaster (Hamilton, Ontario) Universities, using computer-assisted telephone interview (CATI) software (WinCati 4.2, Copyright 1986–2004 Sawtooth Technologies Inc, Northbrook Illinois). Eligible respondents were 18 years or older and living in the household, with no language-mental-hearing barriers. They were queried on whether any of the household members had any of the above 5 food allergies as well as on potential demographic predictors of food allergy. 

To optimize response rates and minimize bias, a maximum of 10 attempts were made to contact households during different days and times between the hours of 9:30 AM to 9:00 PM (local time) Monday through Friday and 10:30 AM to 5:00 PM on Saturdays and Sundays. Households were also advised of our survey a few weeks in advance of the phone call by a mailed information letter. 

The study was approved by the Institutional Review Boards of the McGill University Health Centre and McMaster University.

## 3. Questionnaire

We used a standardized questionnaire developed by Sicherer et al. [5, 12] as in the US, and incorporated questions regarding sesame allergy. The questionnaire was translated into French and back-translated to English. If the eligible household respondent reported that he or she or any family member potentially had a food allergy, the respondent was queried on the history of the most severe allergic reaction, interval between exposure and symptom onset, if medical care was sought and if diagnosed by a physician. The eligible household respondent reported on the household sibship size, annual household income and the respondents' education level, marital status, and country of origin. The eligible household respondent also reported on the ages and gender of all household members. 

## 4. Statistical Analysis

The prevalence of probable food allergy was estimated by including all with a convincing history and/or self-report of a physician diagnosis of allergy. A convincing history was defined as at least 2 mild signs/symptoms or 1 moderate or 1 severe sign/symptom that occurred within 120 minutes after ingestion or contact (or inhalation in those with fish/shellfish allergy). Mild reactions included pruritus, urticaria, flushing, or rhinoconjunctivitis; moderate: angioedema, throat tightness, gastrointestinal complaints, or breathing difficulties (other than wheeze); severe: wheeze, cyanosis, or circulatory collapse [7, 11, 13, 14].

Univariate and multivariate logistic regression analyses were used to estimate the associations between the presence of probable food allergy and potential predictive factors including sibship size, annual household income (low income level defined as an income at which families or unattached individuals spend at least 70% of before tax income on food, shelter, and clothing and is determined according to family size and location), location of household (urban defined as residing in Canadian metropolitan areas or in Canadian areas with a population ≥100,000), province of household (Atlantic provinces, Quebec, Ontario, Prairies, or British Columbia), education level of household respondent (completed college or university), marital status of household respondent (living with partner/married), immigration status of household respondent (Canadian born), ages of each household member (<18 years), and gender of each household member. Possible confounding factors were investigated by comparing univariate to multivariate results. Since the data were collected for randomly selected households rather than randomly selected individuals, all confidence intervals (CI) were corrected in order to account for clustering effects. In addition, a significant proportion of households (38%) did not provide any income data and hence multiple imputation techniques were applied, using all available data, in order to estimate the effect of low income over the largest possible sample. In the case of income-related questions, nonresponse may be due to nonignorable factors. Therefore, two sensitivity analyses were also performed in which the predicted incomes used for imputations were either doubled or halved.

## 5. Results

Three thousand six hundred sixty-six out of 10,596 households contacted responded (34.6% participation rate), of which 3613 completed the entire interview, providing data on 9667 participants. 

Low income and immigrant populations were relatively underrepresented in SCAAALAR (8.9% of households in SCAAALAR were considered low income versus 14.5% in the Canadian population and 14.4% of household respondents in SCAAALAR were immigrants versus 19.4% in the Canadian population) while the SCAAALAR population had a higher proportion of household respondents with postsecondary education when compared to the general Canadian population (60.5% in SCAAALAR versus 32.9% in the Canadian population), the populations were similar with respect to urban versus rural location, province of residence, and marital status (Table 1). 

Peanut, tree nut, and sesame allergy were more common in children (peanut: odds ratio (OR) 2.24 (95% CI, 1.40, 3.59), tree nut: OR 1.73 (95% CI, 1.11, 2.68), and sesame: OR 5.63 (95% CI, 1.39, 22.87)), while fish and shellfish allergy were more common in adults (OR 0.17 (95% CI, 0.04, 0.72) and OR 0.29 (95% CI, 0.14, 0.61), resp.). Tree nut and shellfish allergy were less common in males (OR 0.55 (95% CI, 0.36, 0.83) and OR 0.63 (95% CI, 0.43, 0.91), resp.). Shellfish allergy was more common in urban settings (OR 1.55 (95% CI, 1.04, 2.31)). Higher household education was associated with increased likelihood of allergy to peanut, tree, fish, and sesame although it reached significance only for tree nut (OR 1.9 (95% CI, 1.18, 3.04)). All food allergies were less common in immigrants although large CIs preclude definitive conclusions except for shellfish (OR 0.49 (95% CI, 0.26, 0.95)). Use of multiple imputation for income and the sensitivity analyses did not alter these associations. 

## 6. Discussion

Consistent with other research, our study has demonstrated that peanut, tree nut, and sesame allergy were more common in children, fish and shellfish more common in adults, and tree nut and shellfish allergy less common in males [12, 15–18]. However, ours is the first North American study to examine the influence of education level, immigrant status, and geographic location on food allergy, and we found that most food allergies are more prevalent in the more educated and those born in Canada and shellfish allergy in those residing in urban settings. 

Our results suggest that a higher educational level may be associated with an increased risk of food allergy. These results are consistent with other studies suggesting an increased risk for allergic diseases, including food allergies, in families with higher parental education [19]. However, the mechanisms underlying these relationships are not yet well understood. Given that a higher education level may be associated with changes in family lifestyle [20], the hygiene hypothesis may partially account for these findings. Consistent with the hygiene hypothesis, smaller family size, decreased exposure to pets and livestock, fewer infections during infancy, increased use of antibiotics and vaccinations, and improved sanitation might decrease microbial burden and lead predominantly to a type 2 T-helper cell response which is responsible for triggering allergic disorders [21]. Other factors may also explain this association between education and food allergy. It is possible that more educated parents may be more likely to have followed American Academy of Pediatrics' recommendations [22] regarding the restriction of potentially allergenic foods in early life. This guideline has recently been retracted as research suggests that delayed introduction may promote, rather than reduce, the development of food allergy [23]. Further, educated parents have higher health literacy and may be more likely to consult a physician for suspected food allergies [24]. Hence, the actual prevalence may not be higher in the more educated, but may merely appear increased because of greater likelihood of seeking a diagnosis. 

The observed reduced risk of food allergy in immigrants might be due to genetic differences as well as environmental influences. Recent studies suggest an increased prevalence of allergic diseases commensurate with the length of stay in Westernized countries regardless of age at arrival, sex, or atopic status [25]. Further, it was reported that asthma symptoms in Chinese adolescents were lowest among residents of mainland China, were greater for those in Hong Kong and those who had immigrated to Canada, and were highest among those born in Canada [26]. It was also shown that individuals born in Western countries compared to those born in Asia have a higher risk of peanut and tree nut allergy although the risk for shellfish allergy was unrelated to the place of birth [27]. These observations suggest a crucial role for environmental factors in the pathogenesis of allergic diseases in immigrants. Certain Western dietary habits and lifestyles might contribute to the development of food allergies [28] including omega-3 fatty acids deficiency [29], decreased consumption of fresh fruits and vegetables [30], excess or inadequate vitamin D [31, 32], different food processing methods [33], delayed introduction of foods [10], low-dose cutaneous sensitization to peanut [34], and improved sanitation [35]. It is also possible that immigrants are less likely to consult a physician for a suspected allergy because of lower health literacy [36] and/or lack of a regular family doctor [37], resulting in an apparent, rather than a real decrease in allergy prevalence in this population.

Although a higher prevalence of asthma [38] and eczema [39] in urban settings was previously reported, no population-based studies have examined the association between urban/rural dwelling and food allergy. The higher prevalence of shellfish allergy in urban areas may be attributed to factors related to the hygiene hypothesis including less exposure to parasites and other infections [40], higher use of antibiotics, less exposure to animals [41], less overcrowding (in a house) [42], higher use of processed food [43], and piped water intake (versus spring drinking water) [44]. It is possible that only shellfish allergy was associated with living in an urban setting as it is reported that city dwellers consume more shellfish [45], but not more of the other food allergens. It is also possible that individuals living in urban areas closer to the shellfish sources (i.e., coastal areas) are more likely to have shellfish allergy. However, the association between urban and seafood allergy remains robust even after controlling for coastal proximity through the use of an interaction term (coastal and urban, data not shown). 

Gender differences were observed for tree nut and shellfish allergy. Higher rates of food allergy in postpubertal females are consistent with other studies suggesting that anaphylaxis is more common in adult females [2] potentially due to the effect of estrogens that enhance mast cell activation and allergic sensitization, and progesterone that inhibits histamine release, but potentiates IgE induction [46]. The reduced risk for tree nut and shellfish allergies in males may only be apparent and attributable to a lower rate of physician diagnosis in males as adult males are known to be less likely to have a regular doctor [37]. 

Our study has some potential limitations. As probable food allergy estimates are based on self-report of a convincing history or physician diagnosis, it is possible that the predictors identified in the regression analysis are not valid predictors of a confirmed food allergy (based on the corroboration of history and confirmatory tests). However, given that our probable peanut allergy estimates in Canadian and Quebec children (Canada: 1.68%; Quebec: 1.69%) are consistent with confirmed peanut allergy estimates in Montreal school children (1.63%) [7, 11], we believe that the associations identified for probable allergy are valid for confirmed allergy. Although our participation rate was only 34.6% with an underrepresentation of those of lower socioeconomic status and immigrants, this is consistent with other recent studies [16]. This low participation rate potentially resulted in selection bias with an overrepresentation of those with food allergy. However, given that our estimates for peanut allergy prevalence are consistent with our previous estimates in Montreal school children (as noted above) where the participation rate was 64.2% [7], we anticipate that such a bias is likely to be minimal. Yet, if such a bias does exist, it is likely to make the strength of the association between high socioeconomic status and allergy conservative. We anticipate that among the vulnerable populations, the presence of allergy will increase participation more than it would in the observed population. Hence, if the sample of the vulnerable populations was more representative, the prevalence of allergy in this sample would be even lower and the association between high socioeconomic status and allergy would be even stronger. Other limitations include the availability of data on education and birthplace on only a single household member (i.e., the eligible respondent), and our failure to explore other potential determinants. However, we have data on the education level and birthplace of the principal caregiver of the allergic individual and it is likely that the demographic characteristics of that caregiver play a major role in shaping the household lifestyle and the factors that may contribute to the emergence of food allergies in the household [47]. 

 In conclusion, our results reveal that demographic determinants such as education level, birthplace, and urban dwelling may influence the development of food allergy. Further studies examining the prevalence and pathogenesis of food allergy in vulnerable populations and exploring genetic and other environmental determinants will help disentangle the numerous factors mediating the development of food allergy. 

## Figures and Tables

**Table 1:** Multivariate logistic regression examining association between specific food allergies and respondent characteristics (*n* = 8682*).

	Peanut odds ratio (OR) (95% CI)	Tree nut	Fish	Shellfish	Sesame
Age < 18 yo	2.24 (1.4, 3.59)	1.73 (1.11, 2.68)	0.17 (0.04, 0.72)	0.29 (0.14, 0.61)	5.63 (1.39, 22.87)
Male	1 (0.63, 1.58)	0.55 (0.36, 0.83)	0.96 (0.52, 1.78)	0.63 (0.43, 0.91)	1.04 (0.25, 4.23)
Urban	0.82 (0.5, 1.35)	0.99 (0.65, 1.5)	0.97 (0.51, 1.84)	1.55 (1.04, 2.31)	0.91 (0.18, 4.63)
Immigrant	0.62 (0.28, 1.38)	0.52 (0.25, 1.07)	0.45 (0.14, 1.46)	0.49 (0.26, 0.95)	0.73 (0.08, 6.65)
Postsecondary graduate	1.63 (0.94, 2.85)	1.9 (1.18, 3.04)	1.06 (0.56, 2)	0.69 (0.47, 1.01)	2.43 (0.56, 10.59)**

*This refers to participants providing complete data for the variables in this model; income level was provided by only 5,961 participants.

**Given the small number of sesame allergic individuals, the education variable is university graduate for this allergy.

## Abbreviations

SPT: Skin prick test
CI: Confidence interval
OR: Odds ratio.
